# Some Issues of Occupational Health of Health Care Workers in Central Asia (Review)

**DOI:** 10.29024/aogh.2329

**Published:** 2018-08-31

**Authors:** Baktygul N. Imankulova, Kenesh O. Dzhusupov

**Affiliations:** 1 International School of Medicine, Bishkek, KG

## Abstract

**Introduction:**

In the Global Strategy on Human Resources for Health: Workforce 2030, the World Health Organization (WHO) called for the promotion of decent working conditions for health care workers (HCWs) in all settings. The Central Asian countries (CACs) are low- and middle-income countries with economies past transition and with implemented health care reforms that touch HCWs and occupational health and safety (OHS) at large.

**The aim:**

of the present article is to highlight some challenges in OHS regulation and to highlight some of the latest studies on biological health risks to HCWs in CACs.

**Materials and methods:**

The article reviews 18 journal articles in Russian and 3 journal articles in English published since 2004, 3 dissertations, 10 national statistical sources and profiles, and 5 books.

**Results:**

OHS management systems in CACs are built on the principle of responding to insurance cases, not their prevention. Most labor law standards are declarative in nature because many employers ignore labor protection requirements. There has been no study since 2000 on the working conditions, the physical and chemical health risks or the effects of working conditions, mental and physical loads on the health of HCWs. A few studies were found on work-related infectious diseases (viral hepatitis, HIV and tuberculosis) of HCWs in Kyrgyzstan and Kazakhstan. The latest studies showed a high prevalence of viral hepatitis B, C and G and of HIV infection in HCWs in Kyrgyzstan, with much lower rates in Kazakhstan.

**Conclusions:**

The traditional OHS approach used in soviet times has proven insufficient in CACs with developing market economies. OHS and the health of HCWs in Central Asia is insufficiently studied. The available data indicate a low level of attention to the health of HCWs. Serious studies on OHS in health care settings in this region are needed.

## Introduction

The world faces a global shortage of 12.9 million health care workers (HCWs) in the near future. If no action is taken, this shortage will entail serious health consequences for billions of people in all countries of the world [1]. The 2013 World Health Organization (WHO) report “Universal Truth: No Health without a Workforce” identified a number of reasons for this problem, including unfavorable working conditions of the healthcare workforce [2].

In 2016, for the first time ever, the World Health Assembly admitted the Global Strategy on Human Resources for Health: Workforce 2030. In its twenty-second policy option for all WHO member states, the World Health Assembly called on ministries of health, civil service commissions and employers to promote decent working conditions for HCWs in all settings [3].

It is important to note that effective professional activity of HCWs largely depends not only on their qualifications and the material and technical equipment of health care settings, but also on the state of their own health and working conditions [4].

The European Forum of Medical Associations (EFMA) and the WHO discussed the problem of doctors’ health, drawing attention to doctors’ lack of involvement in managing their own health and failure to give attention to themselves as possible patients [5]. It has been shown that only a fraction of doctors in Europe have their own family doctors, and most diagnoses and prescribed treatments build on their own understandings of their diagnosis.

In most developing countries, occupational health and safety (OHS) is still neglected due to overwhelming socioeconomic and political challenges [6,7,8,9,10,11]. The Central Asian countries are low-and middle-income countries with economies past transition that have implemented health care reforms that touch HCWs and OHS at large [12].

The aim of the present review is to present some challenges in regulating occupational health and safety and to highlight some of the latest studies on biological health risks to health care workers in Central Asian countries.

## Materials and Methods

For the present review, we searched all literature published in English and Russian from 2000 through 2017. We searched electronic databases, such as PubMed, Medline, eLibrary and Google Scholar using the keywords health care workers, occupational health and safety, Central Asia (in course: Kyrgyzstan, Kazakhstan, Uzbekistan, Tajikistan and Turkmenistan). We also searched various hard copies of local journals and books in the libraries of medical universities, the Kyrgyz Ministry of Health and the Kyrgyz and Kazakh national libraries. Round tables were conducted, as well as a review and analysis of national regulations, official statistics, documents of the health ministries and statistical committees of CACs, and materials of international conferences.

Articles included address any aspect of the health or occupational health of HCWs working in five Central Asian countries: Kazakhstan, Kyrgyzstan, Tajikistan, Uzbekistan and Turkmenistan. Other inclusion criteria were the languages of articles published between 2000 and 2017. Articles published in languages other than English or Russian and before 2000 were excluded.

In total, we reviewed 18 journal articles in Russian and 3 journal articles in English, all of which were published in 2004 or later; we also reviewed 3 dissertations, 10 national statistical sources and profiles, and 5 books, and 9 relevant articles from found literature were considered.

## Results

In genearl, national regulations on OHS in CACs are similar [13,14,15,16]. Each country has its own labour codes and laws on occupational safety, which determine the functions and rights of government labour inspectors. Historically in CACs, laws on public health and the sanitary and epidemiological welfare of the population serve as a base for occupational health protection. Among the main legislative acts are regulations on mandatory preliminary and periodic medical examinations, as well as procedures for notification, investigation, registration and reporting of occupational diseases [13,14,15,16].

CACs have similar sanitary norms and rules, though no data was available for Turkmenistan. Upon gaining independence, the CACs joined the International Labor Organization (ILO) and ratified its conventions.

Kyrgyzstan, Kazakhstan, Tajikistan and Uzbekistan share several main principles for OHS systems:

- Employers must preserve the life and health of workers over the benefits and results of any industrial activity;
- Employees are guaranteed the right to OSH;
- The state manages, surveys and controls OHS;
- Occupational accidents, poisonings and diseases must be investigated;
- The legitimate interests of workers who suffer occupational accidents and contract occupational diseases, as well as members of their families, must be protected by mandatory social insurance against occupational accidents and diseases.

In CACs, state inspections, which are under the labour ministries, control the safety conditions in workplaces. The state sanitary and epidemiological centers, which are under the health ministries, conduct surveys to ensure compliance with sanitary norms and to implement measures to prevent occupational diseases.

However, work-related diseases for HCWs are not registered because there is no legislation requiring it. There is no statistical record. The incidence is calculated for the whole population, without separately identifying HCWs. Numbers that are presented are based on the results of a few available studies.

This review considered ten articles on the health and occupational health of HCWs (Table 1).

**Table 1:** Health status and occupational health risk of health care workers in Central Asia.

Author, date	Health status	Occupational health risk factors

Penkina TV et al, 2006	The prevalence of hepatitis C virus (HCV) among Kyrgyz HCWs is higher than that of other Commonwealth of Independent States. The anti-HCV is detected in 7.8% of HCWs.	
Ulmasov R, 2007	The incidence of diseases and days with temporary incapacity of HCWs in Tajikistan increased by 53.2% and 46.3%, respectively, from 2000 to 2005.	
Makhmanurov AA et al, 2013	The rate of chronic viral hepatitis B (HBV) in surgeons was 11.9% and in dentists 11.4%. The incidence of HBV infection was 6.9% in physicians and 6.0% in nurses. The HBV infection was not associated with longer work experience. There was a high infection rate among HCWs with viral hepatitis G (HGV): 14.1%, compared to 1.4% in the general population	The detected rates of infection of HBV, HCV and HGV (as compared to the general population) and of HIV infection of HCWs in Kyrgyzstan, Kazakhstan, Russia and partly in Uzbekistan directly indicate that they are a group at increased risk of parenteral viral infections.
Yrysova MB et al, 2013	Anti-HCV was detected in 15.0% of examined laboratory staff in Kyrgyzstan.	The risk of HCV infection for doctors and nurses who have worked for 10 years and longer was 1.5 times and 3.4 times higher, respectively, than those who have worked less than 10 years.
Smagulov NK et al, 2013	The incidence of medical workers is 6.5 times higher than the incidence rate with temporary disability; 63.2% of HCWs have chronic diseases, 99% of whom are not on dispensary records, and 32% have not undergone preventive medical examinations. The incidence among doctors is higher than among average HCWs.	
Beletsky L, 2013	The total number of HIV-infected HCWs has reached 45 people by 2013. Among them, 13.3% were doctors and 33.3% nurses. Among HIV-infected health workers, 88.9% are women and 11.1% are men.	
Botova OP, 2014	The anti-HCV was detected in 3.19% of examined laboratory personnel in Kazakhstan. The prevalence of HBV and HCV markers among HCWs was 2%. The HCV infection rate among nurses was 2 times higher than among doctors. The most vulnerable to viral hepatitis were laboratory assistants, procedural nurses and surgical nurses.	
Abdylaeva GM et al, 2015		An increase in the number of tuberculosis patients among the population increases the occupational risk to the health of HCWs.
Abdylaeva GM et al, 2017	The incidence rate of tuberculosis among HCWs reached 106.34 per 100,000 in 2014.	Tuberculosis is found on average 10.82 times more often among HCWs of the general medical network compared to the number of tuberculosis cases among employees of specialized TB hospitals.

An analysis of available literature shows there are no studies on working conditions for or physical and chemical occupational health risks to HCWs, and there are no studies on the health effects of working conditions and mental and physical loads on HCWs.

A 2007 study of the health status of HCWs found that as age and work experience increased, there was also an increased frequency of diseases of the respiratory system, as well as an increase in the incidence of hypertension and coronary heart disease [17]. The morbidity rate of HCWs was 6.5 times higher than the incidence rate with temporary disability; 63.2% of HCWs had chronic diseases, of which 99% were not on dispensary records, and 32% had not undergone preventive medical examinations. The incidence among doctors is higher than among average HCWs [17].

The prevalence of immunodeficiency states in medical personnel varied from 25 to 54 per 100 workers in different hospital departments [18].

A few studies found biological risks of infection by viral hepatitis, HIV and tuberculosis for HCWs in Kyrgyzstan and Kazakhstan.

A 2007–2009 study done in Kyrgyzstan showed high infection rates of viral hepatitis B, C and G among HCWs. The rate of chronic viral hepatitis B (HBV) in surgeons was 11.9% and in dentists was 11.4%. The incidence of HBV infection was 6.9% in physicians and 6.0% in nurses. In the study, HBV infection was not associated with longer work experience. There was a high infection rate of viral hepatitis G (HGV) among HCWs (14.1%) compared to a general population (1.4%) [19].

The infection rate of viral hepatitis C (HCV) among nurses was significantly higher (7.6%) than among doctors (5.8%) [28]. High rates of anti-HCV were detected in nurses working in polyclinics (12.5%). Nurses of pediatric departments were more infected than the average staff of polyclinics (14.9%). The risk of HCV infection for doctors and nurses working 10 years or more was 1.5 and 3.4 times higher, respectively, compared to those who have worked less than 10 years [20].

Another study showed that the prevalence of HCV among Kyrgyz HCWs is higher than in other Commonwealth of Independent States. Anti-HCV is detected in 7.8% of HCWs (n = 1636). For comparison, the HCV marker detection rate in Russia is 3.0 to 3.9%, in Ukraine is 3.4%, in Uzbekistan is 6.0% and in Azerbaijan is 6.4% [21].

Anti-HCV was detected in 15.0% of examined laboratory staff in Kyrgyzstan [20] and in 3.19% in Kazakhstan [22].

In Temirtau, the most disadvantaged city in Kazakhstan for so-called haemocontact infections, the prevalence of HBV and HCV markers among HCWs was 2%, which did not exceed the national level [22]. In Kazakhstan, the HCV infection rate among nurses was 2 times higher than among doctors. The most vulnerable to viral hepatitis were laboratory assistants (HBsAg occurs in 3%, HCV in 9.4%), procedural nurses (HCV prevalence 9.1%) and surgical nurses (4.8%).

Despite HBV infection being the most significant infectious occupational hazard in the dental profession [23,24,25], the prevalence of HBV and HCV among dentists in Kazakhstan was lower than the average for all HCWs at 1.89 ± 0.93% and 0.94 ± 0.44%, respectively. The most infected HCWs in Kazakhstan were urologists (HBsAg and HCV made 14.3% of the total number of seropositive results), traumatologists (HBsAg 7.7%), gynecologists (HCV 2.9%) and surgeons (HBsAg 1.8%) [22].

CACs have one of the fastest growing rates of HIV infection [26]. The first reported case of HIV infection of an HCW in Kyrgyzstan was in 2002; by 2013, 45 cases of HIV-infected health workers were reported. Among them, 13.3% were doctors and 33.3% were nurses. Among HIV-infected health workers, 88.9% are women and 11.1% are men [20].

Data on the incidence of viral hepatitis and HIV infection among doctors and nurses in other countries have not been found.

Despite the achievements of modern medicine, the introduction of new therapeutic and diagnostic technologies and highly effective antibacterial and disinfection means, HCWs are at high risk of infection for tuberculosis [27,28].

The incidence of tuberculosis in HCWs is closely related to the incidence of tuberculosis in the general population. Demographic changes in Kyrgyzstan and an increased incidence of tuberculosis in the population have negatively impacted the incidence of tuberculosis in HCWs, because an increase in the number of tuberculosis patients among the population entails a greater likelihood of the infection entering health care facilities [29].

From 2010 to 2014, 201 newly diagnosed tuberculosis cases among HCWs were registered in Kyrgyzstan. The incidence rate of tuberculosis among HCWs reached 106.34 per 100,000 in 2014. Tuberculosis among employees of the general medical network is found on average 10.82 times more often compared to the number of tuberculosis cases among employees of specialized TB hospitals [30]. This indicates a high risk of infection for employees of general medical departments due to insufficient compliance with infection control measures. Among HCWs with tuberculosis, 22 were physicians (10.94% of the total number of patients professionally engaged in health care). Of these, 20 doctors who contracted tuberculosis were from general medical departments. Therapists made up the largest share at 35% (7 cases), followed by surgeons and dentists at 15% (3 cases each), then cardiologists, obstetrician-gynecologists, anesthesiologist-resuscitators, traumatologists, pediatricians, clinical laboratory doctors, and pathologists at 5% (1 case each). There were 14 female (70%) and 6 male (30%) TB patients.

Across the reported incidences of TB in HCWs in all regions of Kyrgyzstan, females predominate because they make up more of the health care workforce. In a 5-year analysis, the number of male HCWs across the country infected by tuberculosis was 24 (11.94%); whereas, the number of female HCWs infected was 177 (88.06%). In total, 142 (70.65%) cases of tuberculosis were identified in HCWs by appeal, and 59 cases (29.35%) were identified during professional examinations. This indicates insufficient quality of conducted medical examinations [30].

## Discussion

A review of national regulations and official reports of CACs showed OHS management systems built on the principle of responding to insurance cases and not their prevention. The analysis of workplace health risks is practically not carried out, mostly due to socioeconomic reasons: an absence or low budget for labor protection inspection and sanitary and epidemiological surveillance. Most attention is paid to compensation measures provided by law in the event of accidents, rather than the prevention of injuries in the first place [31,32].

The main reasons for insufficient OHS are imperfect legislative and regulatory frameworks, failure to fully comply with the requirements of the current legislation on labor protection, insufficient funding of labor protection measures, reduction in the number or total elimination of labor protection services and the absence of a permanent effective system of management and control of OHS [32].

The administrations of medical and preventive institutions do not pay due attention to the OHS of HCWs, underestimating the degree of hazards of the hospital environment as a factor of occupational risk. Doctors and nurses do not attach much importance to this problem, most likely because the policy of safety and health preservation is traditionally applied for patients, not for employees. The lack of attention to their health can be explained by the fact that they are considered professionals who are able to take care of their own health. The task of maintaining the health of the medical staff has fallen on themselves [33]. Therefore, the issues of maintaining safe working conditions and strengthening the health of doctors in the present healthcare system are becoming particularly relevant.

According to the results of the studies given above, the detected rates of infection of HBV, HCV, HGV and HIV in HCWs in Kyrgyzstan, Kazakhstan, Russia and partly in Uzbekistan directly indicate they are a group at increased risk of parenteral viral infections.

Despite HCWs being a risk group for tuberculosis, the tuberculosis epidemic in Kyrgyzstan among HCWs based on clinical manifestations is more favorable than the epidemic among the entire population. HCWs are more likely to detect localized forms of tuberculosis without complications. This is due to the higher socioeconomic standard of living of HCWs compared to the country’s population as a whole, including a large number of migrants, refugees and homeless people. In addition, HCWs are more likely to undergo a fluorographic examination more often than the general population [29].

This review demonstrates there is a need to improve the quality of preventive examinations of HCWs and to strengthen the measures of infection control. A special training program on OHS for HCWs should be developed and provided.

## Conclusions

Occupational health and safety in Central Asian countries seems to be neglected due to overwhelming socioeconomic challenges. The traditional approach to OHS used in the soviet period has proven to be insufficient in CACs with developing market economies. The literature data shows clearly that OHS and the incidence of disease in HCWs in Central Asia is insufficiently studied. The available data indicate a low level of attention to the health of HCWs. Tangible progress in OHS in CACs can be achieved only by effective legislation and linking OHS to the broader context of national development.

Serious studies on OHS in health care settings in CACs are needed to develop effective OHS measures for HCWs and to promote their health.
